# Reference compounds for alternative test methods to indicate developmental neurotoxicity (DNT) potential of chemicals: example lists and criteria for their selection and use

**DOI:** 10.14573/altex.1604201

**Published:** 2016-07-25

**Authors:** Michael Aschner, Sandra Ceccatelli, Mardas Daneshian, Ellen Fritsche, Nina Hasiwa, Thomas Hartung, Helena T. Hogberg, Marcel Leist, Abby Li, William R. Mundy, Stephanie Padilla, Aldert H. Piersma, Anna Bal-Price, Andrea Seiler, Remco H. Westerink, Bastian Zimmer, Pamela J. Lein

**Affiliations:** 1 Albert Einstein College of Medicine, New York, USA; 2 Department of Neuroscience, Karolinska Institutet Stockholm, Sweden; 3 Center for Alternatives to Animal Testing-Europe (CAAT-Europe), University of Konstanz, Germany; 4 Leibniz Research Institute for Environmental Medicine (IUF), Düsseldorf, Germany; 5 Center for Alternatives to Animal Testing (CAAT), The Johns Hopkins University, Baltimore, MD, USA; 6 Doerenkamp-Zbinden Chair for *in vitro* Toxicology and Biomedicine, University of Konstanz, Germany; 7 Konstanz Research School Chemical Biology (KoRS-CB), Konstanz University; 8 Exponent Inc., San Francisco, USA; 9 United States Environmental Protection Agency (US EPA), NHEERL, Research Triangle Park, NC, USA; 10 National Institute for Public Health and the Environment (RIVM), Bilthoven, The Netherlands; 11 Institute for Risk Assessment Sciences, Faculty of Veterinary Medicine, Utrecht University, Utrecht, The Netherlands; 12 European Commission Joint Research Centre, Institute for Health and Consumer Protection, Ispra, Italy; 13 Federal Institute for Risk Assessment (BfR), Berlin, Germany; 14 Neurotoxicology Research Group, Institute for Risk Assessment Sciences (IRAS), Utrecht University, The Netherlands; 15 Axiogenesis AG, Köln, Germany; 16 Center for Research on Occupational and Environmental Toxicology, Oregon Health & Science University, Portland, USA; 17 Department of Molecular Biosciences, University of California, Davis, USA

**Keywords:** neurotoxicity, specificity, test development, AOP, validation

## Abstract

There is a paucity of information concerning the developmental neurotoxicity (DNT) hazard posed by industrial and environmental chemicals. New testing approaches will most likely be based on batteries of alternative and complementary (non-animal) tests. As DNT is assumed to result from the modulation of fundamental neurodevelopmental processes (such as neuronal differentiation, precursor cell migration or neuronal network formation) by chemicals, the first generation of alternative DNT tests target these processes. The advantage of such types of assays is that they capture toxicants with multiple targets and modes-of-action. Moreover, the processes modelled by the assays can be linked to toxicity endophenotypes, i.e. alterations in neural connectivity that form the basis for neurofunctional deficits in man. The authors of this review convened in a workshop to define criteria for the selection of positive/negative controls, to prepare recommendations on their use, and to initiate the setup of a directory of reference chemicals. For initial technical optimization of tests, a set of >50 endpoint-specific control compounds was identified. For further test development, an additional “test” set of 33 chemicals considered to act directly as *bona fide* DNT toxicants is proposed, and each chemical is annotated to the extent it fulfills these criteria. A tabular compilation of the original literature used to select the test set chemicals provides information on statistical procedures, and toxic/non-toxic doses (both for pups and dams). Suggestions are provided on how to use the >100 compounds (including negative controls) compiled here to address specificity, adversity and use of alternative test systems.

## 1 Introduction

### 1.1 DNT testing and test compound selection

Developmental neurotoxicity (DNT) may be broadly defined as an adverse change in the structure or function of the nervous system that manifests after exposure to a chemical during the prenatal or gestational period (Mundy et al., 2015). Notably, the adverse change canmanifest well after the toxicant exposure has ended, a phenomenon referred to as ‘delayed consequence of early life exposure’. This definition raises questions as to the type and magnitude of change considered to a relevant adverse effect. For practical purposes, any statistically significant change may be regarded as an alert for a potential DNT hazard, and then be followed up by more detailed studies. Most considerations of DNT focus on the ‘central nervous system’ but it may be questioned whether the peripheral nervous system, the gastrointestinal nervous system and/or other neural crest-derived tissues should be included in DNT studies.

Traditional approaches for generating data relevant to DNT hazard are largely based on animal testing, according to OECD TG426 and similar standardized protocols developed by national regulatory authorities. Such testing is time- and resource-consuming, which explains why currently only about 200 such studies have been performed with most directed towards pesticides and only a handful focused on industrial chemicals. Even amongst high production volume compounds, only a few have been studied for DNT hazards (Crofton et al., 2012; Rovida et al., 2011). It is also not clear whether these animal testing procedures are sufficiently sensitive to identify all hazardous substances that may affect the developing human brain. For instance, a guideline study on methylmercury, one of the best characterized DNT compounds that targets animals and man, failed to show adverse effects in rats when classical endpoints were condsidered. Only when specific imaging and transcriptomics endpoints were included did this toxicant demonstrate adverse effects on the developing rat nervous system (Radonjic et al., 2013).

Epidemiological studies are an alternate approach for identifying DNT toxicants relevant to man. However, these studies can be particularly challenging due to the time lag between exposure and outcome measurement, and due to the multitude of potentially confounding factors (genetic variability, complex exposures, lifestyle factors, etc.) that affect the complex endpoints studied (e.g. neuropsychological, behavioural or cognitive performance tests). Until 2006, only 6 compounds (lead, mercury, arsenic, PCBs, toluene, ethanol) have been identified unambiguously by epidemiological approaches (Grandjean and Landrigan, 2006); further studies since then have expanded this list to include fluoride, manganese, tetrachloroethylene, chlorpyrifos, DDT, and PBDEs (Grandjean and Landrigan, 2006, 2014). Valproic acid needs to be added to this list based on clinical evidence (Kadereit, 2012; Balmer 2012). Thus, the total number of chemicals (n = 13) identified via clinical/epidemiological studies is rather low to use as a reference chemical set for evaluating or establishing new test systems. Moreover, the epidemiological approach for identifying DNT chemicals provides negligible information as to whether these neurotoxic compounds are direct acting DNT compounds, and which neurodevelopmental processes are perturbed. Such knowledge is critically important for understanding how to use DNT test compounds for the evaluation and optimization of novel test systems. For example, toxicants acting on the thyroid may trigger DNT by decreasing thyroid hormone levels important for nervous system development, but such indirect effects would not be easily detectable in *in vitro* systems based on replicating specific neurodevelopmental processes.

Literature searches recently identified a larger list of DNT compounds that can be used as a reference set for developing and evaluating alternative test systems. A list of 66 compounds with different types of positive and negative controls, and respective comments on mode-of-action was compiled specifically for DNT assay establishment. Amongst this list, only 10 toxicants fulfilled the stringent selection criterium of human evidence. A larger list of about 100 compounds was compiled as part of a published workshop report describing criteria to be applied in DNT test system establishment (Crofton et al., 2011). This list has been complemented with additional background information (e.g. reference to the respective animal studies) and re-published to support the development of high-throughput screening systems (Mundy et al., 2015). This extensive list contains both direct- and indirect-acting compounds, and the quality of the underlying publications shows a large variability. For the present study, a different approach was taken to assemble a list of reference compounds. The main goals were (i) to identify a practical number of chemicals for assay development (about 30 compounds),; (ii) to define clear selection criteria with regards to the published data, and the statistical methods applied to the data reported in these publications; (iii) to document failures to fulfill the selection criteria, and to communicate considerations concerning the use of this compound set for assay development. The intention was not to investigate all potential DNT compounds. For this process, a group of scientists assembled at a workshop developed an initial list of suggested compounds. During the follow-up period, four independent rounds of review by different subgroups of scientists with relevant expertise, resulted in a consensus set of 33 DNT test compounds.

### 1.2 Adverse outcome pathways and fundamental neurobiological processes

Assays (see Box 1 for a glossary) for rapid screening of chemicals with a potential to cause DNT will likely use *in vitro* approaches or alternative models (Bal-Price et al., 2010; Coecke et al., 2007; Smirnova et al., 2014) that are compatible with high throughput screens. The feasibility and utility of such tests is based on the measurement of cellular perturbations relevant to neurodevelopment in humans (Bal-Price et al., 2015b; Kadereit et al., 2012; Lein et al., 2005). The predictive power of these assays will depend on the strength of association between the test endpoints assessed and the neurodevelopmental impairment observed in exposed human populations (or representative mammalian animal models).

In order to facilitate the development and use of molecular and cellular endpoints in predictive assays, the concept of the adverse outcome pathway (AOP) has recently been introduced (Ankley et al., 2010). AOPs are conceptual constructs that link a molecular initiating event (MIE) and an adverse outcome at the level of the whole organism (Tab. 1). A molecular initiating event is the initial point of contact between a chemical and a specific biomolecule that results in a cascade of key events (KE) leading to an adverse outcome (Bal-Price et al., 2015b; Leist et al., 2014). For example, the binding of domoic acid to the glutamate receptor can result in a series of events that result in seizures and memory loss (Bal-Price et al., 2015b; Leist et al., 2014; Watanabe et al., 2011).

**Figure 1:**
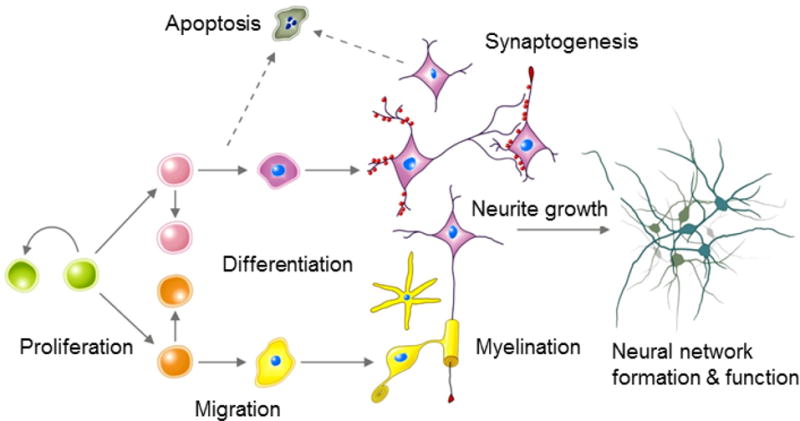
Representation of the key events of neurodevelopment at the cellular level Several fundamental neurodevelopmental processes are absolutely necessary for nervous system development, and therefore well-conserved across species. Moreover, the processes known from *in vivo* studies can be relatively faithfully modeled *in vitro*. It is assumend that DNT exert their toxicity, because they disturb at least one of these processes. Therefore, disturbances of the processes depicted here are KE of AOP relevant for DNT.

In the case of chemicals that cause DNT, most AOPs lack sufficient quantitative features (i.e. quantifiable key event relationships, such as activation thresholds and quantitative time-concentration-effect relationships) to allow specific associations between the molecular initiating event and toxicity manifested at higher levels of biological organization. For this reason, it has been suggested that the first generation of new test methods for developmental neurotoxicity should focus on the assessment of a chemical’s ability to interfere with superordinate ‘fundamental neurodevelopmental processes’ (Lein et al., 2005; Bal-Price et al., 2015a). Studies on neurodevelopment in a variety of invertebrate, non-mammalian vertebrate and mammalian organisms (including man) indicate that the fundamental biological processes of neurodevelopment are remarkably conserved across species (Albright et al., 2000; Cowan et al., 1997; Thomas, 2001; Thor, 1995; Tropepe and Sive, 2003), even though small but distinct differences exist at the mechanistic level, especially the timing of events (Balmer et al., 2014; Smirnova et al., 2015). These ‘fundamental biological/neurodevelopmental processes’ include neural cell proliferation and differentiation, neuronal and glial cell migration, axonal and dendritic outgrowth as well as synapse formation and stabilization, apoptosis and myelination (Fig. 1) (Hoelting et al., 2015; Smirnova et al., 2015; van Thriel et al., 2012). Additional overarching processes, mostly limited to pathological situations reflect different states of glial activation, often termed neuroinflammation (Falsig et al., 2004; Kuegler et al., 2010; 2012; Zerrate et al., 2007). The final outcome of the tightly regulated spatiotemporal execution of these neurodevelopmental processes is the formation of functional signaling networks, and both experimental and clinical studies demonstrate that disruption of the spatiotemporal patterns or magnitude of any of these fundamental processes can significantly alter network connectivity and thus impair neural network function (Tab. 2) (Barone et al., 2000; Berger-Sweeney and Hohmann, 1997; Deoni et al., 2011; Deutsch et al., 2010; Gatto and Broadie, 2010; Jones et al., 2000; Semrud-Clikeman and Ellison, 2009; Smirnova et al., 2015). Because cell-based assays that replicate these fundamental neurodevelopmental processes integrate effects across multiple molecular targets and mechanisms of action, and simple organism-based models additionally integrate effects across multiple cell types and organ systems, these alternative models can “cast a wide net” for detecting chemicals that act through diverse, and potentially unknown, molecular initiating events. Multiple such assays have been developed, e.g. using combinations of human neural cell types, or model organisms like zebra fish, and work with such methods is ongoing to clarify which of the perturbations that are observed show sufficient sensitivity and specificity to be used for predicitions of human adverse effects (Bal-Price et al., 2015b; Bal-Price et al., 2012; Crofton et al., 2011, 2012; Smirnova et al., 2014; van Thriel et al., 2012).

### 1.3 Linking of test systems and apical DNT endpoints

**Figure 2:**
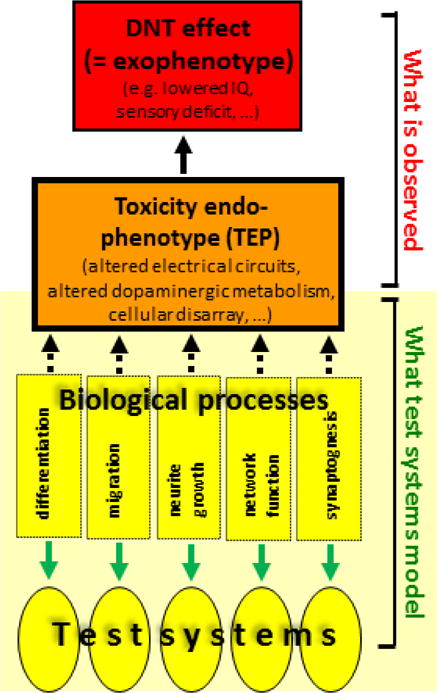
Toxicity endophenotypes For development of relevant model systems, we need approaches for linking the observable DNT effect (= exophenotype; see red box) triggered by a xenobiotic to effects that this compound has in *in vitro* test systems (yellow circles). Toxicity endophenotypes (orange box) form the conceptual link between what is observed in man or experimental animals and on what test systems model. They are a description of the altered biological state of the nervous system (e.g. neuronal disarray in the frontal cortex) *in vivo* that causes the externally observable DNT phenotype (e.g. reduced IQ). Thus, ‘toxicity endophenotypes (TEP)’ describe the altered functional or structural connectivity or responsiveness of parts of the nervous system, triggered by xenobiotics. The TEP results from the disturbance of one or several fundamental biological processes (e.g. neurite growth). Notably, there may be a delay or lag of years between disturbance of a process by a chemical and the observation of DNT effects (dashed arrows linking processes and TEP). Both the setup of model systems and the characterization of tool compounds to validate such systems requires that we establish the following connections: (1) exophenotype to TEP (the exophenotype is the only robust and relevant starting point for identification of DNT compounds known at present); (2) association of TEP with disturbed biological process(es) that led to the TEP; (3) link of *in vitro* test system endpoint to prediction of a disturbed biological process *in vivo*. The fundamental biological processes as such (but not the TEP) may be modeled by alternative test systems. Thus, the test systems are inspired by the biological processes (green arrows), but the outcome of test systems predicts to some extent certain TEP (e.g. inhibited neuronal migration predicts neuronal disarray and/or a deficit in neuronal number in some brain region). In this sense, TEP represent the level of organisation that links *in vitro* test systems for fundamental biological processes to apical DNT endpoints (exophenotypes).

Adverse outcome pathways represent one of several concepts that have been developed to describe the chain of events that link exposure of a biological system to a xenobiotic with the hazard it poses. The concepts differ according to their focus on particular components within the chain of events, and on the intended use of the construct. Quantitative descriptions of the network of cellular events that decide the eventual cell fate are the focus of the ‘pathways-of-toxicity’ approach (Bouhifd et al., 2015; Hartung and McBride, 2011; Kleensang et al., 2014). *In vitro* toxicity testing is the major focus of the ‘biomarkers-of-toxicity’ concept, which concerns the identification of measurable and predictive endpoints that can be applied to model systems. For the purpose of compound selection for DNT *in vitro* assays, the concept of ‘toxicity endophenotypes’ contributes a useful perspective (Kadereit et al., 2012; Balmer and Leist, 2014; Bal-Price et al., 2015a) (Fig. 2). It focuses on fundamental biological processes of relevance to adverse outcomes at the organismal level that can be modeled by *in vitro* systems.

Characteristic adverse outcomes in the field of DNT are cognitive or psychomotor deficits, including reduced IQ, attention deficit, ataxia or various sensory disturbances, in addition to malformations (e.g. spina bifida or microcephaly). They describe external/apical phenotypes that are functionally defined, and which are difficult to model using presently-known *in vitro* systems. Unfortunately, most knowledge on human DNT compounds relates to these externally manifested functional phenotypes (= exophenotypes). For development of relevant model systems, we need approaches to link the ‘exophenotype’ caused by xenobiotic exposure in the intact organism to the effects the compound triggers in *in vitro* test systems. Such associations are the particular focus of the concept of toxicity endophenotypes. Endophenotypes are a description of the altered biological state of the nervous system *in vivo* that underlie the exophenotype. In less theoretical terms, ‘toxicity endophenotypes (TEP)’ describe the altered functional or structural connectivity or responsiveness of parts of the nervous system triggered by xenobiotics, and they represent the level of organization that links *in vitro* test systems for fundamental biological processes to apical DNT endpoints (exophenotypes). All developmental neurotoxicants are expected to affect at least one fundamental biological process *in vivo*, and this would result in an altered TEP. Thus TEP represent a key link between the known effects of DNT chemicals and their effects in *in vitro* systems. (see Tab. 2).

The concept of TEP is also helpful for interpreting test results, evaluating their relevance and choosing endpoint-specific tool compounds in such systems. In this context, it is important to distinguish between the TEP (a state that is assessed *in vivo*) and the disturbed biological processes that led to it (and which may be assessed *in vitro*). For instance, a disarray of cells in a certain brain region may be the result of inhibited migration, altered patterning or even reduced neurite outgrowth that prevents axons from reaching appropriate target regions, and therefore results in apoptotic elimination or aberrant wiring.

### 1.4 Practical implications for the choice of positive-control compounds

The theoretical dissection of various associations relevant for the interpretation of DNT test system data (Exophenotype ←→ endophenotype ←→ biological processes ←→ test systems) has important practical significance, for instance by identifying research gaps and showing needs for further biological information. An important knowledge gap for DNT toxicants is the link between disturbed fundamental biological processes and TEP. This essential piece of information is difficult to obtain, as there is often a delay between chemical disturbance of a neurodevelopmental process and the DNT manifestation. Without knowledge on this link, it is not possible to define positive control toxicants for *in vitro* test systems that reflect only one of few biological processes relevant for DNT (Westerink, 2013). This has three important consequences. The first is that evaluation of test system performance (predictivity) with ‘known’ DNT chemicals is problematic using the standard approach of statistical correlation. The first type of misinterpretation are false negatives. If a test system does not react to a given DNT compound, the test system would be interpreted as lacking sensitivity, even though many DNT compounds would correctly show no effect in any given test system. In these cases, compounds cause their toxicity by affecting fundamental biological processes that are not captured by the test system in question. For instance, test systems that evaluate neurite extension or synapse formation would not be expected to react to methylazoxymethanol (MAM), an established DNT chemical (Penschuck et al., 2006) that affects precursor cell proliferation. A second type of misinterpretation/pitfall refers to examples of false positives that occur if a test system reacts to a compound that does not cause DNT in humans (*in vivo*) by altering the biological process evaluated in this system. For instance, if MAM, which as indicated above is a compound that specfically affects dividing cells, shows an effect in a test system of synapse formation, this would most likely be a false positive, from the point of view of mechanistic toxicology. However, it needs to be noted that it could be a true positive affecting a target different from DNA that has simply not yet been identified in *in vivo* systems due to their low sensitivity and high noise. Practical example for such a case are found when examining litereature on direct effects of chlorpyrifos on biological systems *in vitro*. For instance, voltage -gated calcium channels are inhibited by the parent compound, while the well-established inhibition of acetylcholine esterase is more sensitive to the oxon metabolite (Meijer et al., 2014a,b).

The second consequence is that sets of compounds other than ‘gold standard DNT chemicals’ are required to initially evaluate the performance of *in vitro* test systems. Such chemicals should affect the known biology and mechanisms of the test system in defined, and, preferentially, specific ways. These compounds, here termed ‘endpoint-specific controls’ or ‘endpoint-specific reference compounds’ (Tab. 3), are in many cases not known to be associated with DNT. Therefore, the evaluation of the usefulness and relevance of the test system would not be possible through correlation of chemical’s effects *in vitro* vs. *in vivo*. It rather needs to be based on biological plausibility. One of the experimental approaches to this issue is the identification of the signaling processes governing the test system and their mechanistic relevance to signaling processes known to control the corresponding biological processes *in vivo*. The relevance and role of such signaling processes could be tested with sets of mechanistically-defined tool compounds. This would help to link the underlying biology of the test system to TEPs that are produced by genuine DNT compounds.

A third consequence is that the major usefulness of a set of positive DNT compounds lies in the establishment and evaluation of a test battery, rather than individual assays. The serious limitations that apply to individual tests (see first consequence) do not apply to a test battery that aims to cover the majority of DNT adverse effects. Compounds that are defined as gold standard positive controls should be identified as hits in the test battery (or an associated integrated approach to testing and assessment (IATA)). If they are not identified in the test battery, they would be correctly classified as false-negatives. Vice versa, negative controls should not be identified as hits, or they would be classified as false positives. Thus, a set of control compounds would be useful to evaluate an IATA approach (Bal-Price et al., 2015b; Rovida et al., 2015), and at the same time they would be useful in guiding the establishment of a test battery and for identifying data gaps to be filled using tests of higher sensitivity for specific compounds.

## 2 Endpoint-specific control compounds

### 2.1 The concept of endpoint-specific control compounds

Assays (test methods) for DNT propose the use of both *in vitro* models based on neural cell cultures and alternative (non-mammalian) species as test systems. This guarantees that there will be a wide variety of measurements used to detect a change induced by a test substance, ranging from molecular (e.g. RNAs, proteins) to biochemical (e.g. neurotransmitters and their receptors) to morphological (e.g. cell size, shape or motility) to functional (e.g. locomotor activity, receptor function, electrophysiological properties). These measurements, regardless of the format, should assess an endpoint related to a fundamental neurodevelopmental process. A particular test system may allow for assessing multiple endpoints related to the same neurodevelopmental process. For example, the endpoint of proliferation can be assessed using both biochemical measurements of the amount of DNA and the morphometric assessment of cell numbers. As part of the setup and evaluation of a new test method, it should be demonstrated that measures for an endpoint are robust, reproducible (Miller, 2014; Poland et al., 2014) and accurate, and that the dynamic range within the test system is characterized. Moreover, different ways of measuring the same endpoint should yield similar results (consistency of readout). The next crucial step is the demonstration that a chemical-induced change in the biological endpoint can be detected. To describe this phase of assay evaluation, the concept of endpoint-specific controls has been introduced. Endpoint-specific controls (also termed ‘endpoint-selective controls’ or ‘mechanistic tool compounds’) (Crofton et al., 2012; Kadereit et al., 2012; Leist et al., 2010) are chemicals that are known to reliably alter the endpoint of concern in a particular test system. Ideally, endpoint-specific control chemicals would be used to demonstrate both an increased and decreased response. They are selective in that within a known concentration range, the chemical will alter the primary test endpoint (e.g. precursor cell proliferation) without affecting general test system characteristics, including measures of cell viability. To continue with the example of proliferation, an endpoint-specific control would decrease (or increase) the measures of DNA and cell number within a test system in the absence of a change in cell viability. For neural cell proliferation, such chemicals include those with a known mechanism (e.g. the DNA polymerase inhibitor aphidicolin or the spindle poison taxol) or those where the mechanism is unclear but for which there is substantial literature evidence demonstrating selectivity (e.g. cadmium for certain systems).

Endpoint-specific controls are typically used in the initial evaluation of assay performance. In this sense, they are considered as “positive control” chemicals since they should be chosen based on prior knowledge that they alter the endpoint of concern under similar conditions using an established measurement. For example, studies from multiple laboratories have demonstrated that the MEK (MAP kinase kinase) inhibitor U0126 decreases neurite length in PC12 cells in a concentration-dependent manner (Kano et al., 2002; Liu et al., 2006). Thus, U0126 was used as an endpoint-specific control to determine if biochemical assessment of GAP-43 was a suitable measurement for neurite outgrowth in PC12 cells (Das et al., 2004). In the case where the test system is capable of producing an endpoint response in both directions, endpoint-specific controls for both an increased response and decreased response are desirable. For example, neurite outgrowth in PC12 cells can be increased above that measured under standard culture conditions by treatment with the IP3 kinase inhibitor C5 (Eva et al., 2012). Once an endpoint-specific control for a particular test system has been identified and characterized, it can be used as a “within-assay” or “within-plate” reference control during chemical testing. This internal control helps to identify plate-to-plate or test-to-test variability and to establish historical response levels. This is done by including one or more replicates containing a concentration of the endpoint-specific control known to produce a measurable response in the endpoint of interest without altering other outcomes. Moreover, such reference measurements can be used to define acceptability criteria for test results (on a per-plate or per-day basis).

### 2.2 Selection of endpoint-specific controls

The selection of endpoint-specific control compounds should be based both on the fundamental neurodevelopmental event being assessed and the test system being used. Prior knowledge of developmental neurobiology may identify signalling cascades required for the biological process evaluated in the test system and/or suggest pharmacological or drug-like chemicals that specifically target those signaling pathways. These “mechanistic tools” (Kadereit et al., 2012) would have a high probability of a positive effect within the context of a test system for a specific system’s endpoint. However, knowledge of the “mechanism” of a chemical is not a prerequisite for identifying an endpoint-specific control if there is sufficient evidence showing selective effects on an endpoint within a test system. The following criteria should be considered when identifying chemicals to be used as endpoint-specific controls:

#### Peer-reviewed data

Of primary importance is the previous demonstration in the peer-reviewed literature that a chemical alters the endpoint within a particular test system. Reliability of the effect is demonstrated by showing the full concentration-response behaviour, providing evidence for the selectivity of the chemical for the endpoint of interest compared to other possible outcomes (e.g. cytotoxicity, metabolic competence, etc.). Demonstration of mechanistic consistency is highly desirable, e.g. demonstration that a kinase inhibitor indeed inhibits the target kinase in the relevant concentration range (in which it affects the systems endpoint) in the given test system. Studies using a single concentration or without a concurrent measure of general cell health do not provide sufficient data to identify endpoint-specific controls.

#### Demonstrated effects in multiple test systems

The demonstration that a chemical meets the criteria listed in criterion A (above) in more than one test system (e.g. different cell types) or under multiple conditions (e.g. different cell culture media or different periods of exposure) increases confidence in its application as an endpoint-specific control. Data for the same chemical should ideally be available from multiple laboratories.

#### Knowledge of chemical mechanisms

Chemicals with a known target (molecular initiating event) or known actions at various levels of biological organization increase reliability for a selective effect on a particular neurodevelopmental endpoint. Knowledge of the signaling pathways underlying a fundamental neurobiological process in a given test system can help to identify potential endpoint-specific controls. Sometimes test system development will require acquisition of this biological knowledge, by screening of known pathways or identification of new pathways by broad screening approaches and use of omics methods.

#### Chemical causes same qualitative effect in vivo

Some endpoint-specific controls may cause the same qualitative effect in an *in vitro* test system and *in vivo*, i.e. it may affect the fundamental neurodevelopmental process that is modelled in the *in vitro* test in a live developing mammal. The congruence of results from standard (*in vivo*) and alternative test methods (*in vitro/lower model organisms*) increases confidence that the chemical is selectively acting on a fundamental neurodevelopmental endpoint. However, this is not a mandatory criterion, as several good endpoint-specific controls may not be active *in vivo*, due to metabolism, toxicokinetic reasons or off-target toxicity. Based on these criteria, endpoint-specific control compounds for fundamental neurodevelopmental processes have been compiled (Tab. 4).

### 2.3 Selection of negative controls

Once an assay has been established and has been shown to react to endpoint-specific controls, some basic evaluation of specificity is important. This requires compounds that have no effect in the test system. Such negative controls do not perturb the respective fundamental neurodevelopmental process, or its underlying signalling pathways. The ideal negative controls can be defined as chemicals that are biologically (pharmacologically) active in other systems, but are not expected to have an effect on the endpoints of the test system under evaluation. To demonstrate absence of effect, a concentration should be used that shows a significant effect in other test systems.

In practice, it is sometimes difficult to identify pharmacologically potent compounds devoid of any DNT effect. In such cases, the simplest type of negative controls are compounds that do not cross the cell membrane (such as mannitol). Groups of chemicals with good potential as negative controls are nutrients (e.g. ascorbic acid), chemicals that target other organ systems (e.g. the liver toxicant paracetamol), or chemicals with a known target (molecular initiating event) that is not expressed in the test system (e.g. the proton pump inhibitor omeprazol) (Kadereit et al., 2012). Alternatively, drugs that are recommended for use in pregnancy are an important resource, but all of them require individual evaluation. Few suggestions for negative controls for evaluation of DNT assays have been compiled (Tab. 4). For these compounds, no peer-reviewed papers reporting on their developmental neurotoxicity could be identified. Preference is given to compounds that have been actively tested for DNT, but were found experimentally to be negative.

### 2.4 How to deal with specificity

Many published test systems reach high levels of sensitivity for some known DNT compounds, but little information is available on specificity. This issue is directly related to the topic of compound selection for DNT test systems, as specificity of a test system is defined as the capacity to classify negatives correctly, i.e. specificity correlates with a low rate of false positives. Thus, selection and testing of negatives is an essential step in the optimization cycles of test system establishment. This task is not trivial, as it is not sufficient to simply select compounds for which there is currently no evidence that they trigger DNT.

Three considerations are important for the selection of good negative controls for specificity testing: (i) First, the biological process modeled in a test system is not the same as the phenotype resulting from exposure to a DNT chemical *in vivo* (see TEP above). Therefore, ‘non-DNT chemicals’ may specifically affect a test system (see endpoint-specific controls above), and the task to find real negatives is often difficult, and it needs to be determined for each test system; (ii) The second reason is the potential for interaction of test endpoints. For instance, viability and neurite growth are two endpoints in a given test system, but they are not independent of one another. For example, some xenobiotics may affect a specific test endpoint (neurite growth) indirectly by acting on cell viability. Thus, such compounds would appear as positive hits, although they are true negatives with respect to the primary biological process (neurite growth) examined in the test system. The most frequent of these phenomena is decreased cell viability by a ‘nonspecific’ test compound, which subsequently influences the test endpoint(s) of primary interest. Therefore, care needs to be taken that overall reduced cell viability or decreased cell survival is not interpreted as an effect on differentiation, neurite growth, migration or synaptic connectivity (all of which may also be affected because viability is reduced). A straightforward approach to this problem is testing of compounds only at concentrations determined to not cause cytotoxicity in that test system. However, unambiguous definitions on how non-cytotoxic concentrations should be determined do not exist at present. To eassess the specificity of a test system for direct-acting DNT compounds, it is necessary to select a second group of negative control compounds, i.e. nonspecific controls known for their general cytotoxicity (Kadereit et al., 2012; Leist et al., 2010). The concentration ratio of these compounds concerning specific (e.g. neurite growth) and nonspecific (e.g. cytotoxicity) test endpoints can be used to define a prediction model for test specificity (Krug et al., 2014; Stiegler et al., 2011); (iii) The third problem is related to toxicokinetics (including drug metabolism). Several compounds would (based on their biochemical activity) affect fundamental neurodevelopmental/biological processes relevant to DNT, but they are not recognized as DNT compounds in the literature or by *in vivo* testing, as they do not reach the fetus or the central nervous system at the doses used. Such compounds would be scored as false positives in *in vitro* assays, with respect to *in vivo* effects, but they would in fact be true positives with respect to the biology tested in the assay. Thus, a task for the future would be to provide background (toxicokinetic) information on such effects and compounds.

## 3 Selection of high-quality DNT reference compounds

### 3.1 Selection procedure and rules

A group of neurotoxicology experts from government, academia and industry convened in Konstanz, Germany, (October, 2011) to identify chemicals for potential use as positive controls for developmental neurotoxicity. The selection was based on two major principles: (a) the list of chemicals was intended to be exemplary, and not exhaustive. The initial selection of candidates did not follow a defined screening process or data base search algorithm, rather it was based on the subjective recall of the experts of frequently-quoted litereature or their own work. The aim was to establish a list of 20–30 compounds useful for assay development and evaluation, and compounds with solid evidence for DNT activity may not have been considered; (b) after compilation of a primary list, compounds were vetted using pre-defined criteria (Box 2). The purpose of the selection criteria was to ensure that the selection process was based on scientifically sound studies. Moreover, the goal was to increase the likelihood that the selected positive controls act as direct developmental neurotoxicants, and that adverse effects are not the indirect consequence of maternal toxicity. The supplementary material contains extensive information on the low-effect-levels (LOELs) and no-effect-levels (NOELs) for offspring, maternal toxicity and the DNT endpoints affected.

Candidate compounds that largely failed to meet these criteria were eliminated from the list. Compounds that met many of the criteria were retained, and the criteria that were not met are flagged. In general, the supporting documentation for these compounds derives from published animal studies, but in some cases, human epidemiological evidence based on multiple studies was available as additional supportive evidence. Most of the evidence on human effects is derived from authoritative reviews (Grandjean and Landrigan, 2006, 2014) that compiled available evidence for DNT effects in a systematic way. However, complete weight-of-evidence evaluations are available for all compounds. For example, there is still controversy in the field as to the relevance of DNT effects of chlorpyrifos at human exposure levels (Burns et al., 2013; Li et al., 2012; Mount et al., 2009).

The list of DNT reference chemicals (Table 5 and supplementary material) should be considered a sample list of positive control chemicals that have the potential of causing developmental neurotoxic effects in animals at some dose level, which may or may not be relevant to human exposure levels. Of the 33 compounds listed, the majority (n = 29) overlap with the more extensive list assembled by scientists from the EPA (Mundy et al., 2015). The non-overlapping references suggested here are the pesticide lindane, the recreational drug 3,4-methylenedioxy-N-methamphetamine, and the groups of perfluorinated aliphatic compounds comprising perfluoro-octanoic acid (PFOA) and perfluoroactane-sulfonic acid (PFOS).

Note, the list of reference DNT compounds presented here requires an evaluation of its fit-for-purpose by end-users, and this implies elimination or addition of compounds for specific purposes or additional literature searches on specific compounds within the list. A future step may be the compilation of systematic reviews on each of the compounds, with respect to the weight of evidence that they are developmental neurotoxicants in animals. For instance, here only positive evidence for DNT effects was considered. It was neither weighed against the entirety of the available literature of a given compound (which may also include negative studies), nor did we consider that there may be a publication bias (with negative findings less likely to be published). A systematic review would also provide information on whether a parent compound acts directly as developmental neurotoxicant as well as the role of metabolism in toxifying or detoxifying the parent compound. This consideration is pivotal for chemical use in *in vitro* systems as well as alternative species models in which metabolism can vary from that of humans. For instance, chlorpyrifos may need to be converted to chlorpyrifos-oxon (Yang et al., 2008), heroin may fail to show effects in systems that lack deacetylases that catalyze the formation of the final toxicant morphine, and 1-methyl-4-phenyl-1,2,3,6-tetrahydropyridine (MPTP) will fail to show any effect, unless it is metabolized by astrocytic monoamine oxidase to 1-methyl-4-phenylpyridinium (MPP^+^) (Efremova et al., 2015; Schildknecht et al., 2015).

### 3.2 Use of the DNT compound set

After setup of a test, and evaluation of its technical performance and reproducibility on the basis of endpoint-specific controls, the next steps involve, amongst others: (i) gathering information on the predictivity of the test; (ii) establishment of a prediction model; (iii) introducing additional endpoints and/or adjusting parameters for increased rate of data collection or higher throughput; (iv) identification of biomarkers, measurable key events or signaling mechanisms that may be used to build or optimize other test systems, or translation to human studies; and (v) characterization of the MOA of known DNT toxicants to evaluate which AOP key events are reflected by the test system, and for which types of mechanisms the test is applicable. For such activities, a set of highly relevant (i.e. high confidence that they indeed trigger DNT *in vivo*) control compounds is essential.

For instance, one traditional way to evaluate predictivity would be to split the pool of DNT compounds into a training set and a testing set. Using the data generated with the training set plus negative controls, a prediction model would be established. The validity of this model, and its performance (accuracy, specificity, sensitivity) would then be tested by blinded measurement of the testing set. In a variation of this approach, the splitting of the compounds into training and test sets would be done in silico in many, or in all combinatorily possible ways, after all compounds have been tested.

Introduction of new endpoints or identification of biomarkers (Krug et al., 2014; Stiegler et al., 2011; Zimmer et al., 2012) requires the availability of a relevant set of test compounds that allow correlation studies from one system or from one endpoint to another. To an even greater extent, the same holds true for identifications of general toxicity mechanisms (Fritsche et al., 2005; Gassmann et al., 2010, 2014; Langeveld et al., 2012; Lein et al., 2007; Waldmann et al., 2014; Yang et al., 2014; Zimmer et al., 2011a) or for development of toxicant classifiers (Krug et al., 2013b; Rempel et al., 2015), as the selected compounds are the main anchoring point of such studies.

## 4 Challenges encountered during the search for reference compounds

### 4.1 Research bias

Two examples have been selected here (phenytoin, isotretionin, see sub-chapter below) to illustrate the challenges of selecting reference compounds for DNT, based on criteria of high quality data, and statistically sound human or animal studies.

Concerning animal data, the studies are often old, and the design and reporting standards are not up to current demands for documenting a gold standard reference compound. Some studies only show a (non-significant) trend or a possibility that a compound is a DNT toxicant. Nevertheless, such data may have important implications for further handling of such compounds. Such initial findings may prevented further studies to establish statistical significance of the effects and to meet the quality standards established here for compound selection. This may have been due to several reasons. For instance, institutional or regulatory approval for animal experimentation are hard to obtain if an experiment is mainly confirmatory of previous findings (even though these are not of high quality). Another reason is that funding is hard to obtain for confirmatory studies that differ from earlier findings mainly in statistical power and rigor of design.

Concerning human data, a similar situation is frequently observed, i.e. initial weak evidence makes it difficult to obtain further, more definite evidence. The major reason here is that once a potential hazard has been documented, measures will be taken to reduce the risk, i.e. human exposure to the compound in question is kept to a minimum. Therefore, obtaining epidemiological data on compounds with a suspected DNT hazard is particularly difficult. A way around the problems described above could be the increased use of a battery of alternative methods that is sufficiently evaluated for its performance and predictivity.

### 4.2 Phenytoin and isotretinoin exemplify challenges in obtaining high quality literature data

This situation is demonstrated by two suspected DNT compounds, phenytoin and isotretinoin. They did not fully fulfill the statistical and documentation criteria identified in Box 2, but they were included (see details below) in our compound collection (Tab. 5) with indication of the limitations of the available published literature.

Diphenylhydantoin (phenytoin) is a sodium channel blocker used as an anticonvulsant antiepileptic drug. In the literature, a malformation, called ‘fetal hydantoin syndrome’ is observed in children exposed to phenytoin during fetal development. Fetal hydantoin syndrome is associated with cerebellar malformations and psychomotor dysfunction after intrauterine exposure (extensively reviewed by Vorhees (Vorhees, 1994)). Several animal studies are suggestive of hydantoin being a DNT toxicant. Described effects range from impaired synapse function (Forcelli et al., 2012) and neurodegeneration (Asimiadou et al., 2005) to general neurotoxicity (Hatta et al., 1999). However, the studies fail to fulfill the full set of criteria, defined by the workshop participants for a DNT reference compound (Box 2, Tab. 5). There are also several reports that suggest phenytoin is a human DNT toxicant, but a review (Nicolai et al., 2008) covering 56 studies concerning teratogenic effects of antiepileptic drugs, concluded: *“The identified studies do not allow definite conclusions. The possibility of neurodevelopmental delay, behavioural disorders, or learning disabilities as an outcome of in utero exposure to AEDs needs to be considered seriously. The literature however does not provide evidence for a valid risk estimate”*.

Isotretinoin is one of the isoforms of retinoic acid (usually the generic name retinoic acid refers to the all-trans isoform, while isotretinoin has one cis-bond (position 13). It is the active ingredient in the highly effective antiacne drug Accutane and is suspected to cause depression and suicide in adults and neonatal malformations. From 1982 to 2006, more than 2,000 isotretinoin users became pregnant. Amongst them, a high frequency of spontaneous or elective abortions was observed. As of 2002 — the year generic Accutane was approved — the FDA had received reports of 172 babies born with a congenital defect or anomaly after maternal use of Accutane^2,3^. They quote: *“Accutane is clearly a potent human teratogen that causes malformation of the central nervous system, cardiovascular system and facial structures”*. This is, however, not supported by animal studies that meet the quality criteria set out here. The reason is interesting and very instructive. Already in the 90s it became clear that the teratogenicity of some compounds depends on pharmacokinetics (Nau, 1986). Isotretinoin (Nau, 2001) is one of the drugs that shows negligible effects in mouse and rat (Kochhar and Penner, 1987; Kamm, 1982), while in monkeys (Fantel et al., 1977) and (possibly) humans, isoretinoin can cause great disturbances of embryonic development. It is assumed that most effects of isotretinoin (13-cis-retinoic acid) are mediated by isomerization to all-trans-retinoic acid. Concerning this metabolic prerequisite, the situation has been described as follows: ‘*The insensitive species (rat, mouse) eliminate the drug rapidly through detoxification to β-glucuronide; also, placental transfer is limited in these species. On the other hand, in sensitive species (primates), the drug is predominantly metabolized to the active 13-cis-4-oxo-retinoic acid; placental transfer is more extensive here’* (Nau, 1986)*.*

The two above examples clearly demonstrate the difficulties with compiling a definite and exhaustive list of DNT chemicals. Likely there are other compounds that could be included in the list, and, there are likely many compounds that are DNT toxicants, but that lack sufficient animal or human data to be considered gold standard reference compounds for test evaluation.

### 4.3 Examples of other compounds not considered here

The test set presented here may be complemented by additional compounds as determined by personal preference or scientific needs. They may be selected from a recently-published 100 compound collection or from newly emerging publications on DNT (Mundy et al., 2015). In all cases, it is advisable to apply the criteria delineated in Box 2 to additional compounds. Amongst the more reently discussed copounds with a potential to cause DNT is paracetamol (Brandlistuen et al., 2013; Liew et al., 2014; Viberg et al., 2014), but it is not clear yet whether this effect is direct or whether it requires metabolic activation. There are also indictions that the food-borne non-proteinogenic amino acid BMAA affects neurodevelopment (Karlsson et al., 2015). The same is true for acrylamide, a chemical generated from amino acid precursors during food processing (Duarte-Salles et al., 2013; Pedersen et al., 2012). However, more information regarding specificity is required; for example, acrylamide’s effects on head circumference and brain weight may also be indirect consequences of toxicity. Also not included here is the developmental toxicant cyclopamine (Cooper et al., 1998), a plant ingredient with broad developmental effects that is listed amongst the endpoint-specific controls for neurodifferentiation assays.

## 5 The path forward

### 5.1 How to get more mechanistic information on DNT compounds?

One of the major problems for developing and evaluating DNT assays remains the fact that there is a paucity of information regarding the effects of DNT compounds on fundamental neurobiological processes in humans. This precludes an evaluation of test predictivity based solely on the correlation of its results with *in vivo* findings (Leist et al., 2012). One way forward would involve the three following activities: (a) obtaining more knowledge on modes of action of DNT chemicals by profiling them in a broad set of well characterized and robust *in vitro* test systems (Behl et al., 2015; Zimmer et al., 2014; Daneshian et al., 2016; Hirsch et al., 2016; Pallocca et al., 2016); (b) optimizing *in vitro* test systems, by using endpoint specific controls and already well-characterized DNT compounds; (c) using steps (a) and (b) in an iterative fashion to optimize test systems and test methods.

The path forward also involves increased greater understanding of the biology underlying the test systems, understanding why certain compounds work or do not work, and learning exactly why DNT reference compounds work in some systems, but not in others. This process requires mechanistic interventions, follow-up on pathways-of-toxicity and studies of groups of related compounds (Dreser et al., 2015; Krug et al., 2013a; Krug et al., 2013b; Zimmer et al., 2011a). Most likely, test systems will need to be characterized by many different analytical approaches to derive the needed information. Limitation to a single, toxicologically-relevant endpoint will not be sufficient in this establishment and optimization phase of a test system.

### 5.2 How to deal with adversity vs adaptation

For all *in vitro* assays, it is difficult to distinguish between changes that are linked to adverse effects *in vivo*, and alterations that are adaptive or counter-regulators (Blaauboer et al., 2012). An overall solution to this challenge will be a major issue for the future. In the context of compound selection, a few points deserve immediate attention and action. The first and foremost is ‘concentration’. The questions of specificity and adversity cannot be linked to compounds as such, but only to a ‘compound at a given concentration’ (Waldmann et al., 2014; Daston et al., 2014). Although this appears trivial, it has hitherto been scarcely considered when specificity and sensitivity of an assay have been evaluated. In addition, most screens have up to now been performed at fixed compound concentrations that are not related to the pharmacological potency of the compounds screened. A change of this practice has been suggested for the ESNATS test battery (Pallocca et al., 2015; Zimmer et al., 2014), for which initial concentrations for testing have been based on a biological/mechanistic rationale. In addition, for many omics studies the chosen concentration is anchored to a biological effect (e.g. maximum non-cytotoxic concentration). In practice, the task of determining which concentrations are meaningful and correspond to *in vivo* effects is not trivial, and they can be quite difficult to determine (Westerink, 2013). A future useful step for the field would be the drafting of a consensus document addressing the feasibility of basing concentrations for DNT testing on reverse pharmacokinetic modelling (Bosgra et al., 2014). One of the approaches for defining adversity would be based on measuring concentration-dependency of many endpoints in the system and relating these dependencies to the concentration known to be associated with adverse effects *in vivo*. Another useful approach would be to not only rely on measurements at a defined time point at the end of the incubation, but to follow the temporal evolution of changes in the system in the absence versus presence of test compounds (Dreser et al., 2015).

### 5.3 How do we link test systems in vitro to DNT in vivo?

The usual evaluation of a test system addresses three domains: reproducibility, biological relevance and correlation with *in vivo* data (= predictivity). Determination of predictivity is only possible to a limited extent because of the lack of large numbers of well-characterized DNT chemicals, thus, more focus will need to be put on the first two domains (Basketter et al., 2012; Leist et al., 2012). A significant problem with the existing *in vitro* test systems for the identification of developmental neurotoxicants is the lack of explicit guidance on how to standardize DNT endpoints. Clear quality control procedures would be required for *in vitro* models to produce results comparable across laboratories, and with the ultimate goal to use data for regulatory purposes. To address biological relevance, several different approaches may be combined (Alepee et al., 2014; Hartung et al., 2013; Smirnova et al., 2015; van Vliet et al., 2014). One approach is directly related to the selection of test compounds: the understanding of the response to tool compounds, and mechanistically consistent responses to chemically-related compounds would be helpful to evaluate the biological relevance of the test system. Similar types of information for *in vivo* DNT, including information on the temporal evolution of the damage, would be very helpful.

### 5.4 How can the information obtained using DNT reference compounds be applied to develop more predictive assays?

The selection of chemicals that can serve as endpoint-specific controls will facilitate quality control and standardization of *in vitro* models. Systems would be expected to react in a predictable manner to positive and negative controls before they can be used further for chemical testing. Moreover, the study of DNT reference compounds compiled here will create an important data base for the characterization of new test systems, and for elucidating whether the ‘molecular machinery’ present in a cell system is capable of responding to known developmental neurotoxicants as expected.

The understanding of the pathways-of-toxicity/AOP induced by DNT reference chemicals could serve as a template to design assays that will be based on the key events that determine outcome. Such assays may have reduced complexity and higher throughput, and they would directly address selected AOP of relevance for DNT. To apply the AOP concept to DNT evaluation, a clear description of the measureable parameters is required to study each key event (Bal-Price et al., 2015a, 2015b; Edwards et al., 2016; Perkins et al., 2015; Tollefsen et al., 2014).

With respect to the selection of chemicals and their characterization in DNT *in vitro* test systems, applying the AOP concept will provide important information for the development of structure-activity relationships (SAR) and “read-across”, i.e., using information from one chemical to predict the effects for another one, that is structurally related. This will allow grouping and ranking of chemicals according to their modes of action and potency (Dreser et al., 2015; Ramirez et al., 2013).

Based on comparing data generated across multiple diverse test systems, the most sensitive endpoints and the most reliable test systems could be selected for a ‘test battery’ as the basis for an IATA (see Box 1). One of the steps forward in this direction would be establishment of high-throughput screening assays. The data from such assays could be used for chemical prioritization, screening of chemicals for further *in vivo* testing (Bal-Price et al., 2012; Crofton et al., 2012, 2014; Judson et al., 2014), obtaining information on mixtures of compounds, integration of the data by systems toxicology methods (Hartung and McBride, 2011; Sauer et al., 2015), and reducing reliance on *in vivo* testing for regulatory decision-making.

## Supplementary Material

supplemental material

## Figures and Tables

**Tab. 1:** Examples of events relevant for adverse outcome pathway (AOP) linking exposure to DNT chemicals to human toxicity An AOP represents a series of measurable key events (KE) with biologically plausible connections. They connect a molecular initiating event (MIE) to an adverse outcome (AO) in an individual. The AOP is a concept that provides a framework for organizing knowledge about the progression of toxicity events across scales of biological organization. Here examples are given for MIE, for KE (on the cellular and organ level), and for AO, i.e. the manifestation relevant for man, that may be triggered by DNT chemicals. The cellular KE correspond to fundamental neurodevelopmental processes as detailed in Fig. 2

Molecular initiating events (MIE)	Key events (KE) – cellular responses	Key events (KE) – organ responses	Adverse outcomes (AO)
Modulation of the function of ion channels; inhibition of assembly or disassembly of cytoskeletal elements; inhibition of key enzymes (e.g. acetylcholine esterase or receptor tyrosine kinases); inhibition of the mitochondrial respiratory chain; inhibition of transporters on the cell membrane or organellar membranes; inhibition or stimulation of nuclear receptors; inhibition of cell-cell or cell-matrix contacts; inhibition of DNA synthesis; modulation of epigenetic processes (e.g. histone modifications or DNA methylation); etc.	Neural precursor proliferation; migration; gliogenesis; neuronal differentiation; neurite growth (axons, dendrites); synaptogenesis; oligodendrogenesis; myelination; programmed cell death; neuroinflammation; etc.	S. nigra dopaminergic neuron degeneration; Hippocampal dentate gyrus neuronal dysarray; Hypomyelination in periventricular white matter; lissencephaly; microcephaly; holoprosencephaly; altered EEG pattern; attenuated prepulse inhibition; altered contents of serotonin in a brain region; altered threshold to seizure-inducing treatment; etc.	Reduced learning ability; shortened attention span; autism spectrum disorders; reduced memory and executive functions; anxiety; reduced mood control and stress resilience; etc.

**Table 2:** Apical *in vivo* endpoints of DNT translated to DNT endpoints *in vitro* *In vivo* studies use various methods to evaluate DNT. These can be roughly classified as anatomical measures (e.g. morphology, histopathology) or as functional measures (e.g. motor, sensory and cognitive function). These methods assess various outcomes (e.g. malformations detected by anatomical measures) or changes (increase/decrease) in functional parameters. Each of these outcomes derives from changes in cellular biology (e.g. altered apoptosis, cell migration or cell proliferation may lead to size differences of brain regions). The cell biological changes may be modeled by *in vitro* or alternative test methods.

Methods *in vivo*	Outcome	Cell Biological Causes
**Gross morphology**	Brain measures↑↓ Brain parts missing Malformation	→ Proliferation, apoptosis → Proliferation, differentiation → Proliferation, migration, differentiation
**Histopathology**	Necrosis Pyknosis Neuronal degeneration Astrocytosis Layer thickness ↑↓	→ Cytotoxicity → Apoptosis, necrosis → Neurotoxicity → Glia proliferation, GFAP content → Proliferation, migration, myelination, cell death
**Morphometry**	Layer thickness ↑↓ Morphology	→ Proliferation, migration, myelination → Proliferation, migration, differentiation
**Learning/Memory/Motor Activity**	**↑↓**	→ Synaptogenesis → Network formation → Specific death of neuronal subpopulations → Myelination

**Table 3:** Tool compounds/endpoint-specific controls for DNT test systems Assays were classified according to the basic biological process they are modeling (left column). The literature was then screened for compounds that elicited robust positive responses in respective in vitro test systems. These compounds were classified according to their inhibiting or activating effect on the baseline or control readout. For compounds that interfere with cellular differentiation, this one-dimensional classification was not attempted. For practical purposes (choice of positive controls useful during assay setup), the table contains not only classical endpoint-specific controls but also chemicals/toxicants with unclear mode of action, but with a robust effect on the targeted endpoint. They were considered useful to evaluate the technical performance of the test system with respect to the endpoints measured. For each compound, the original literature documenting its effect on the targeted endpoint is indicated.

	Inhibitory	Stimulatory
**Migration**	methylmercury^7^^,^^8^, PP2^7^^,^^8^, AG1478^8^, PD98059^8^, SU6656^8^, SP600125^7^, pertussis toxin^7^, lead acetate^7^, triadimenol^7^, thimerosal^7^, semaphorin3A^7^, valproic acid^7^, CK-666^7^, cytochalasin D^7^, 3-methylcholanthrene^9^, 7NI^10^, ODQ^10^	albumax^7^, phorbol myristate acetate (PMA)^8^
**Proliferation**	aphidicolin^11^^,^^12^^,^^13^, cadmium^11^^,^^12^^,^^13^, cytosine arabinoside^11^^,^^12^^,^^13^, 5-fluoroacil^11^^,^^12^^,^^13^, methylmercury^13^	epidermal growth factor^4^
**Synaptogenesis**	mevastatin^15^, potassium chloride^15^	
**Network activity**	bisindolylmaleimide^16^	domoic acid^17^
**Neurite outgrowth**	methylmercury^14^^,^^18^^,^^19^^,^^20^^,^^21^, U0126^14^^,^^18^^,^^19^^,^^20^, bisindolylmaleimide I^14^^,^^15^^,^^18^, lithium^14^^,^^15^^,^^20^, sodium orthovanadate^20^^,^^22^^,^^23^, retinoic acid^14^^,^^18^, brefeldin A^20^, flavopiridol^20^, cycloheximide^2^, paraquat^2^, diquat^2^, rotenone^2^, nocodazole^2^, colchicine^2^, vincristine^2^, narciclassine^2^	Y-27632^20^, HA-1077^2^, blebbistatin^2^
**Oligodendrocyte differentiation**	PBDE-99^24^, PBDE-47^24^	thyroxin^25^, PCB 118^25^
**Differentiation** (compounds known to alter this process (adversely) in one of many possible ways)	methylmercury^1^^,^^2^^,^^3^^,^^4^, mercury chloride^5^, valproic acid^2^^,^^3^, trichostatin A^3^, retinoic acid^6^, lead acetate^6^, cyclopamine^6^, bone morphogenetic protein (BMP)4^3^	

The numbers behind the compound refer to the literature references as follows:

1 (Zimmer et al., 2011b),

2 (Krug et al., 2013b),

3 (Balmer et al., 2012),

4 (Moors et al., 2009),

5 (Moors et al., 2010),

6 (Zimmer et al., 2011a),

7 (Zimmer et al., 2012),

8 (Moors et al., 2007),

9 (Gassmann et al., 2010),

10 (Tegenge et al., 2011),

11 (Mundy et al., 2010),

12 (Culbreth et al., 2012),

13 (Breier et al., 2008),

14 (Harrill et al., 2011a),

15 (Harrill et al., 2011b),

16 (Robinette et al., 2011),

17 (Hogberg and Bal-Price, 2011),

18 (Radio et al., 2008),

19 (Radio et al., 2010),

20 (Stiegler et al., 2011),

21 (Parran et al., 2001),

22 (Harrill et al., 2010),

23 (Mandell and Banker, 1998),

24 (Schreiber et al., 2010),

25 (Fritsche et al., 2005).

**Table 4:** Suggestions for negative tool compounds A set of potential negative controls has been assembled, and experience from multiple assays will be needed to further refine this list. Although absence of activity cannot be proven, compounds with a very high likelihood to not affect DNT assays are found amongst sugar derivatives, solvents and polymeric compounds that do not enter cells. These types of relatively trivial negative controls mainly provide an indication of assay robustness and background noise levels, but do not provide much information regarding assay specificity. Another group of potentially negative control compounds are those with defined pharmacologic effects or other measurable bioactivity that are unlikely to trigger DNT or to affect fundamental neurodevelopmental processes. However, compounds for which this information is known are not available for every test system. Notably, any compound has the potential to affect biological systems at high enough concentrations. Therefore, specific compounds are useful as negative controls only if used at appropriate concentrations. This may be the concentration known to be bioactive in other systems (e.g. clinically-observed plasma levels for drugs), the highest non-cytotoxic concentration or the highest concentration used for any positive control (e.g. 100 μM – 1 mM), as higher chemical concentrations are unlikely to occur in any in vivo situation. Note that compounds like nicotine may be good negative controls for some assays, e.g. cell migration, but endpoint-selective positive controls for other assays, e.g., neural network assays. Importantly, the absence of a drug’s specific target in a test system (e.g. warfarin), does not mean that there is not another, less characterized (or unspecific) target, that still leads to effects on test endpoints.

Compound	Comments	Literature
Anthracen	Polycyclic aromatic hydrocarbon; may act via Ah receptor, but has no target in many human DNT/NT test systems	1
3-Imino-propionitrile	Neurotoxicant, requiring metabolic activation. Low toxicity if test system lacks activating enzymes	2
Metoclopramid, amitryptilin, ibuprofen, metoprolol, sumatriptan, amoxicillin, diphenhydramine	Drugs that are acceptable during pregnancy	10
Pomalidomide	Thalidomide analog, no DNT up to 200 μM	3
Omeprazole/warfarin	Drugs with primary target only in stomach/liver; low likelihood to have DNT effects	4^,^ 5
Captopril, dabigatran	Drugs with extracellular targets	–
Solvents: dimethylformamide, DMSO, glycerol	Generally low toxicity up to mM range	–
Sugar (derivatives): sorbitol, lactose, mannitol, glucosamine, diethylene glycol	No pronounced bioactivity, sometimes not entering cells, tolerated to mM level;	
Belongs to “trivial” controls (low usefulness for specificity calculations) with solvents	–	
Glyfosate	Pesticide tested negative for DNT; low cytotoxicity	–
Dinotefuran	Neonicotinoid pesticide without DNT effects in many systems (may however effect neuronal network assays)	6
Fipronil	Pesticide tested clearly negative for DNT; may be cytotoxic at > 10 μM; may have indirect effects through cramp induction (zebrafish)	7
Deprenyl	Antidepressant/parkinsonian drug, inhibitor of monoamine oxidase-B (1 mM range)	–
Acetaminophen/paracetamol	Negative in most systems up to mM levels, but has been discussed as *in vivo* DNT toxicant	8^,^ 9
Saccharin	Artificial sweetener, very low toxicity	–
Trolox, zVAD-fmk	Water-soluble vitamin E analog; caspase inhibitor (usable at 100 μM)	–
Deferoxamine mesylate	Iron chelator, tolerated at mM levels	–
Furosemide, verapamil, levetiracetam, statins, seroquel, naloxon, atropine. ursodeoxycholic acid, tiotropium	Drugs with low likelihood to affect DNT test systems, due to their well characterized side effects and mode of action (may have direct effects on neural networks, though)	
RU38486, propylthiourcil, testosterone…	Hormone modifiers little relevant to *in vitro* DNT test system targets	–

The numbers behind the compound refer to the literature references as follows:

1 (Pei et al., 2015),

2 (Ryan et al., 2016),

3 (Mahony et al., 2013),

4 (Gill et al., 2009),

5 (Ekman et al., 1985),

6 (Sheets et al., 2016),

7 (Krug et al., 2013a),

8 (Burdan, 2003),

9 (Reel et al., 1992),

10 (Niebyl and Simpson, 2008).

**Table 5:** Compounds triggering DNT *in vivo* An initial list of compounds was collected from the literature by way of subject expert suggestions. This list was intended to be exemplary and not exhaustive or even complete. In a second step, each compound was scrutinized for published literature supporting its DNT activity. The criteria described in Box 2 were applied to evaluate supporting literature (supplementary excel file). As an additional criterion, we used ‘strong evidence for DNT effects in humans’ as documented by well-recognized meta-analysis or well powered studies (column ‘Hu’, for human evidence). Compounds were retained in the list when at least two publications from two different laboratories in support of their DNT activity were identified. Published studies were categorized into one of four certainty groups: a) animal study that meets all criteria as described in Box 2 (score 3); b) study describes human data with statistically representative populations or study represents meta-analysis of human findings (score: 3), c) animal study in which one criterion is not met (score: 2); d) animal study in which 2–4 criteria were not met (score 1). For the classification of papers, criteria 5 and 8 as described in Box 2 were not included, but they are indicated for transparency. For the assessment of the certainty of the developmental neurotoxic effects of the selected compound, the scores were averaged. Compounds with a score of 2.5 or higher are presented in green, compounds with a score of 1.5–2.5 are presented in light green. Compounds with lower scores were eliminated. The superscript numbers (explained in Box 2) for each publication indicate the selection criteria not met. The comment field gives an indication on the endpoints used in the studies. If different types of endpoints were used they are indicated in the sequence of the listed publications, separated by semicolon.

Compound	Reference	Additional comments	Hu
Arsenic	5; 6^e,f;^ 7^f,h^	Behavior	2
Cadmium	8^e,i;^ 9	Behavior	
Chlorpromazine	10^e^; 11^f,h,i^	Behavior; seizure threshold	
Chlorpyrifos	12; 13	Brain cholinesterase inhibition; brain weight and morphometry	3
Cocaine	14; 15^h,j;^ 16^h,j^	Human; behavior + morphology	
Dexamethasone	17^e,f,i^; 18^e,f^	Behavior; behavior, brain chemistry; human: cortisol values, stress response	19
Diphenylhydantoin (Phenytoin)	20^i^;^21^^i^	Behavior; behavior, eye opening	
Domoic acid	22^e^; 23; 24^e,f,h,i^	Conditioned place preference, activity; memory, behavior; neurochemistry	
Ethanol	25; 26^i,j^; 27	Human: behavior; behavior, learning; attention; human: morphology	4, 28
Haloperidol	29^e,f,h^; 30^h;^ 31^h^	Behavior/cognitive	
Heroin	32^e,h,i^; 33^e,h,i^; 34^h,i^	Human: behavior	4
Hexachlorophene	36^e,h,j^; 37	Human: neuropathology; vacuolation of brain white matter	35
Ketamine	38^e,j^; 39; 40^h^	Motor activity, learning, memory; increased apoptosis; behavior, spatial learning	
Lead	41^f,i^; 42^e,i^; 43^f,I,j^	Human; behavior; mRNA expression, brain enzymatic activity; brain chemistry	1
Lindane	44^f,I,j^; 45^e,h^	Behavior	
MAM	49^h,j^, 50^f^, 51^h,i,j^	regional brain weight; increased innervation, neurochemistry; brain morphometry	
Maneb	52^e,i^, 53^h^	Behavior; behavior, morphology (*in vivo* cell count)	
Manganese	46^e,f,h,i^, 47^e,h^, 48^f,j^	Behavior, brain chemistry	3
MDMA	54, 55^h,i^	Behavior; neuropathology; human: cognition; human: mental/motor development	56, 57
Methanol	58^h,I,j^, 59^h^, 60^e,h,i^	Behavior	
Methyl mercury	61, 62^e,f,h^, 63^i^	Human; behavior; behavior; neurobiochemistry, transcriptomics	1
MPTP	64^e,h^, 65^e^	Behavior, brain neurochemistry; behavior	
Nicotine	66^e,h^, 67^h^	Behavior	
Paraquat	68^e,f^, 69^h^	Behavior; brain neurochemistry	
PBDE	70^e^, 71^h^, 72^e,h^	Behavior; behavior, pharmacologic challenge; electrophysiology	3
PCB	73, 74	Human: Behavior, brain morphometry; behavior	1
Perfluorate – PFOA	75^e,f^, 76^e,h^	Behavior	
Perfluorate – PFOS	77^e,i,j^, 78^e,i^, 79^e,j^	Hippocampus structure; behavior, motor activity, learning, memory,	
Terbutaline	83^h^, 84^e^	Behavior; behavior, neuroinflammation	
Toluene	85^e^, 86^e^	Behavior; brain weight	1
Trans retinoic acid	80^i^, 81^i,j^, 82^h^	Behavior; behavior; motor coordination, learning, brain morphology	
Triethyl-tin	87^j^, 88^j^	Behavior, brain cell count; brain weight, myelin basic protein	
VPA valproic acid	89, 90^e^	Behavior	

References:

1 (Grandjean and Landrigan, 2006),

2 (Tolins et al., 2014),

3 (Grandjean and Landrigan, 2014),

4 (Yolton et al., 2014),

5 (Martinez-Finley et al., 2009),

6 (Chattopadhyay et al., 2002),

7 (Rodriguez et al., 2002),

8 (Baranski, 1984),

9 (Ali et al., 1986),

10 (Robertson et al., 1980),

11 (Golub and Kornetsky, 1975),

12 (Johnson et al., 2009),

13 (Maurissen et al., 2000),

14 (Mactutus et al., 2011),

15 (Kabir et al., 2014),

16 (Lu et al., 2012),

17 (Hossain et al., 2008),

18 (Benesova et al., 1999),

19 (O’Connor et al., 2013),

20 (Weisenburger et al., 1990),

21 (McCartney et al., 1999),

22 (Doucette et al., 2003),

23 (Levin et al., 2005),

24 (Dakshinamurti et al., 1993; Lucchi et al., 1983),

25 (Oshiro et al., 2014),

26 (Lucchi et al., 1983),

27 (Brys et al., 2014),

28 (Fryer et al., 2012),

29 (Watanabe et al., 1985),

30 (Wolansky et al., 2004),

31 (Rosengarten and Quartermain, 2002),

32 (Lasky et al., 1977),

33 (Yanai et al., 1992),

34 (Wang and Han, 2009),

35 (Shuman et al., 1974),

36 (Ulsamer et al., 1975),

37 (Itahashi et al., 2015),

38 (Fredriksson et al., 2007),

39 (Paule et al., 2011),

40 (Zhao et al., 2014),

41 (Petit et al., 1992),

42 (Hu et al., 2008),

43 (Reddy et al., 2007),

44 (Johri et al., 2007),

45 (Rivera et al., 1990),

46 (Lown et al., 1984),

47 (Kristensson et al., 1986),

48 (Deskin et al., 1981),

49 (Sullivan-Jones et al., 1994),

50 (Cattabeni et al., 1989),

51 (de Groot et al., 2005),

52 (Sobotka et al., 1972),

53 (Thiruchelvam et al., 2002),

54 (Broening et al., 2001),

55 (Thompson et al., 2012),

56 (McElhatton et al., 1999),

57 (Singer et al., 2012),

58 (Stern et al., 1997),

59 (Infurna and Weiss, 1986),

60 (Aziz et al., 2002),

61 (Elsner et al., 1988),

62 (Sakamoto et al., 2002),

63 (Radonjic et al., 2013),

64 (Ochi et al., 1991),

65 (Fredriksson et al., 1993),

66 (Levin et al., 1993),

67 (LeSage et al., 2006),

68 (Fredriksson et al., 1993),

69 (Thiruchelvam et al., 2002),

70 (Viberg et al., 2003),

71 (Dufault et al., 2005),

72 (Dingemans et al., 2007),

73 (Yang et al., 2009),

74 (Sable et al., 2006),

75 (Johansson et al., 2008),

76 (Onishchenko et al., 2011),

77 (Zeng et al., 2011),

78 (Butenhoff et al., 2009),

79 (Johansson et al., 2008),

80 (Nolen, 1986),

81 (Holson et al., 1997),

82 (Coluccia et al., 2008),

83 (Owens et al., 2011),

84 (Zerrate et al., 2007),

85 (Hass et al., 1999),

86 (Burry et al., 2003),

87 (Freeman et al., 1994),

88 (O’Callaghan et al., 1983),

89 (Vorhees, 1987),

90 (Schneider and Przewlocki, 2005).

## Box 1 Glossary for assay definition and setup

### Test methods vs test systems

#### Test system

Cellular (or biochemical) system used for a test method (e.g. “proliferating hESC”, or “neuronally-differentiating PC-12 cells”, or “organotypic brain slices”). The term is often used interchangeably with “in vitro system”, or sometimes also termed “biological model”. The test system is only one component of a test or ‘test method’. Good performance of a test system does not imply good functioning of a test method. Acceptability criteria for test systems (e.g. at least 75% of the differentiated cells staining positive for nestin under control conditions) are different from acceptability criteria for the test method using the test system (e.g. inhibition of differentiation by a specified positive control by at least 35%, and alteration of normal differentiation by a defined negative control by less than 10%)

#### Test method

A procedure based on a test system, used to obtain information on the biological effects of a substance. A toxicological test method consists of four major components (i.e. test system, exposure scheme, endpoint, prediction model), and it produces a test result (information regarding the ability of a substance or agent to produce a specified biological effect under specified conditions). The term is used interchangeably with “test” and “assay” in the literature. A test method can have several analytical endpoints.

#### Prediction model

Prediction model: a formula or algorithm (e.g., formula, rule or set of rules) used to convert the results generated by a test method into a prediction of the (toxic) effect of interest. Also referred to as decision criteria. A prediction model contains four elements: (1) a definition of the specific purpose(s) for which the test method is to be used; (2) specifications of all possible results that may be obtained, (3) an algorithm that converts each study result into a prediction of the (toxic) effect of interest, and (4) specifications as to the accuracy of the prediction model (e.g., sensitivity, specificity, and false positive and false negative rates). In this context, ‘Data Interpretation Procedure (DIP)’ is of interest. It signifies any algorithm for interpreting data from one or more information sources. The output of a DIP is typically a prediction (e.g. prediction of skin sensitisation potential from peptide binding data and/or chemical structure).

#### Acceptance criteria

Criteria defined before performing an assay to determine whether it is “valid”, i.e. whether the data can be used. Typical issues of acceptance criteria comprise: ‘has the actual run or plate of the test method functioned (e.g. are the endpoint values for PC and NC in the right range)’, ‘is the test method performing within the desired range of variability (e.g. are the standard deviations of PC and NC in the right range)’. Note: acceptance criteria can also be defined for an ‘analytical endpoint’ or for a ‘test system’.

#### Endpoint

The biological or chemical process, response, or effect assessed in a test system by a specific analytical method/assay, e.g. “viability” as measured by LDH-release, expression of a marker as measured by PCR, or beating of cardiomyocytes evaluated by an imaging system. Note that each endpoint may be assessed by different ‘analytical methods’. For instance, ‘viability’ may be assessed by LDH-release, resazurin reduction, cell counting or measurement of ATP. “Differentiation” may be measured by PCR quantification of a differentiation marker or by morphometry (e.g. beating of cardiomyocytes evaluated by an imaging system.

#### Analytical endpoint

An endpoint of a test system (e.g. proliferation, or differentiation, or viability) may be quantified by different analytical methods (measurement endpoins). It is important to distinguish such analytical endpoints (referring to the methods used) from (test systems) endpoints that refer to the biological concept evaluated.

#### *In vitro* system

This term has various meanings in the litereature, i.e. it is little defined. It is sometimes used to signify a cell/tissue culture system used as the basis for the development of a test method. In this sense it corresponsds to a test system (as above). (Note: In biochemistry, the term is often used for cell-free systems, as opposed to cellular (living) systems. Cell culture assays, i.e. *in vitro* assays in a toxicological sense, are often called “*in vivo* systems” in biochemistry).

#### Assay

This term is used in a broader or narrower sense depending on the field, similar to ‘test method’. In a narrower sense, ‘assay’ can refer to an analytical procedure (e.g. protein determination, PCR). In a wider sense, ‘assay’ is used interchangeably with ‘test method’. A classical example is the Ames assay, which comprises a complex test system of growing and plating bacteria under different conditions together with an analytical procedure based on the counting of colonies.

### Reference compounds and statistics

#### Positive/negative control (PC/NC)

A PC is a compound or condition that triggers a response, i.e. a change of the endpoint from baseline in a predicted direction and to a certain specified extent. A NC for a ‘test method’ is a compound or condition that should not trigger a response, i.e. it should not change the endpoint from baseline. The performance of PC and NC can be used to define ‘acceptance criteria’ of a test.

#### Endpoint-Specific Controls

Chemicals known to reliably and consistently alter the endpoint of a test system at a mechanistic level. These are also referred to as ‘endpoint-selective controls’ or ‘mechanistic tool compounds’. This would the first set of compounds used during test system setup to obtain information on the biological/toxicological behaviour of the test system and its dynamic range.

#### Training Set Chemicals

This set should include chemicals known (preferably from in vitro systems) to reliably elicit a response, or no response, with respect to the endpoint of interest. The goal of using this set is proof-of-concept that the test method can rapidly and efficiently screen moderate numbers of chemicals with reasonable predictivity. A training set of chemicals can be used to optimize an assay (test method), to set acceptability criteria, and to build a prediction model.

#### Testing Set Chemicals

This set would be used to validate and possibly improve the prediction model. For DNT, this set should include chemicals known to affect (and also some that definitely do not affect) in vivo developmental neurotoxicity endpoints The goal of using ‘testing set chemicals’ is also to demonstrate the ability to test larger numbers of chemicals.

#### General cytotoxicity (GC)

The term is used when a compound triggers cell death that is not specific for the cell type used in the assay but would occur in most cells at the same concentration and within a similar time frame. For many test methods it is important to measure specific adverse effects that occur at concentrations below those triggering cell death in the test system. Therefore, the verification of test conditions not triggering GC is important for many tests.

#### Unspecific controls (UC)

Unspecific controls (UC): often refers to compounds displaying GC. For some test systems, it is sufficient to work with PC and NC. For other test systems, it is important to demonstrate a difference between compounds that act specifically, and compounds that lead to changes of the endpoint because they trigger GC. For instance, a test may be designed to determine the metabolic fingerprint of cell cycle blockers. Such a test would require the examination of UC and the comparison of their profile with PC compounds.

#### Highest non-cytotoxic concentration (HNCC)

Highest non-cytotoxic concentration (HNCC): the highest concentration of a compound that does not trigger GC. The HNCC is important, as it allows the detection of specific adverse effects with highest likelihood. It defines the highest concentration to be used in test systems examining particular toxic effects independent of GC. Testing at concentrations higher than the HNCC may lead to artifacts.

#### Replicates within one experiment

These are also called “technical replicates” and can take two different forms: A: the repeated performance of an analysis on the same sample, e.g. duplicate PCR, Western blot or FACS determinations. B: the determination of an endpoint from more than one culture well, with all these wells being incubated in parallel/on the same day/in the same experiment.

#### Independent experiments

These are also called “biological replicates” and should not be confused with technical replicates in different dishes. A biological replicate is a separate experiment, i.e. on another day, with independent cell batches, new test solutions, etc. A biological replicate can comprise several technical replicates.

#### Robustness/Ruggedness

Is a measure of a methods’ capacity to remain unaffected by small variations in method parameters and environmental conditions. Testing of robustness provides an indication of a test’s reliability during normal usage. Sometimes a distinction is made between robustness and ruggedness. The latter focuses on the reproducibility of the test results obtained for identical samples under normal test conditions that underlie unintentional changes (room temperature, source of human sample material, lot variation of reagents, operator-dependent variables, weather conditions, etc.). Robustness testing would explore the insensitivity of a test to deliberate variations in the test environment or setup (incubation time, temperature, cell passage number, sample storage, cell density, type of culture dish, etc.

#### Dynamic Range

Determination of the extent of measurable change that can be detected for a DNT endpoint and whether both increases and decreases from untreated control can be measured.

### Test concepts

#### Fundamental biological process

In the context of DNT, this refers to ‘fundamental neurodevelopmental process’. These processes include precursor cell proliferation, neuronal and glial cell differentiation and apoptosis, synaptogenesis and myelination, and are also termed ‘key biological processes’ or ‘key neurodevelopmental events’. They need to be distinguished from signalling events or more basic mechanisms, in that ‘fundamental biological processes’ represent a higher (superordinate) level of organization, that comprises many signaling mechanisms and targets of molecular intiating events. They are ‘fundamental’, as failure of any of them may result in DNT. Importantly, these processes can be modeled using *in vitro* test systems, and each such ‘test system’ has the advantage of capturing (identifying) many different toxicants acting by different molecular mechanisms. Note: fundamental biological processes are not to be confused with key events (KE) in an AOP.

#### Molecular initating event (MIE) and key events (KE)

A molecular initiating event is the initial point of contact between a chemical and a specific biomolecule that results in a cascade of key events (KE) leading to an adverse outcome.

#### Adverse outcome pathways (AOPs)

Adverse outcome pathways (AOPs): conceptual constructs that link a molecular initiating event (MIE) to an adverse outcome at the level of the whole organism. The AOP links existing knowledge along one or more series of causally connected key events (KE) between two points — a molecular initiating event (MIE) and an adverse outcome (AO). AOP are not compound-specific, but a theoretical construct applicable to multiple compounds.

#### Toxicity endophenotypes (TEP)

Altered functional or structural connectivity or responsiveness of specific regions of the nervous system as a consequence of exposure to xenobiotic(s). TEP represent the level of organization that links *in vitro* test systems for fundamental biological processes to apical DNT endpoints *in vivo* (exophenotypes).

#### Integrated Approach to Testing and Assessment (IATA)

Integrated Approach to Testing and Assessment (IATA): an approach based on multiple information sources used for hazard identification, hazard characterization and/or safety assessment of chemicals. An IATA integrates and weighs all relevant existing evidence and guides the targeted generation of new data, where required, to inform regulatory decision-making regarding potential hazard and/or risk.

## Box 2 Criteria used to select chemicals as positive DNT controls

Note: the letters refer to the superscripts in table 4.

- At least two peer-reviewed papers from two independent groups of investigators reporting evidence of DNT. For human data: only positive meta-analysis of multiple studies.
- DNT evidence from *in vivo* mammalian models with exposures during gestation and/or lactation (either direct pup exposure or exposure to the dam) prior to weaning; robust human epidemiological data also considered.
- Exposure is to the test chemical itself and not to formulations, its metabolites or mixtures (Shafer et al. 2005, Shafer and Crofton, 2011).
- Outcomes were neurobehavioral, neurophysiological, functional (including pharmacologic responses), brain anatomic or pathology findings not due to acute effects of exposure. Findings based solely on neurochemical, gene expression or biochemical endpoints were excluded from consideration.
- The statistical unit (e.g. individual pups or litters) is reported. For animal studies with gestational or early postnatal exposure (either lactational or direct dosing), the litter is the experimental unit (De Sesso et al., 2009, Holson et al., 2008). Violation of this criterion was accepted but flagged, as it was not always possible to distinguish because of poor study design and/or poor reporting standards.
- Minimal sample size is reported and is at least n=6 (i.e. 6 litters/dose group for gestational or early postnatal exposure studies).
- Studies were not included if the route of exposure was intracerebral injection. Preference was given to studies using human-relevant routes of exposure (i.e. oral, dermal, inhalation).
- Studies should be based on at least 2 dose-levels; some violation of this criterion was allowed but flagged, since single dose studies in one publication may often build on previous experience of the group with multiple doses, with subsequent studies based on the most appropriate dose.
- Relationship of maternal toxicity versus DNT: Ideally, DNT should occur at lower doses than maternal toxicity. Studies in which maternal toxicity occurred at the same dose as DNT, or where this was not reported, were flagged.
- Relationship between DNT and general toxicity: ideally, DNT should occur at lower/same concentrations than general toxicity. Studies in which general toxicity/mortality occurred at the same concentration as DNT, or in which this was not reported, were flagged/highlighted. Studies where this relationship was not reported were also flagged.

## Footnotes

2 http://www.drugwatch.com/accutane/side-effects.php

3 http://www.cdc.gov/mmwr/preview/mmwrhtml/00000310.htm
